# Causes of interruption in enteral nutrition in CCU patients - experience from an indian hospital

**DOI:** 10.1186/2197-425X-3-S1-A580

**Published:** 2015-10-01

**Authors:** A Kar, VRA Rao, A Datta, A Ahmed

**Affiliations:** Medica Superspecialty Hospital, Medica Institute of Critical Care, Kolkata, India

## Introduction

In Critical Care Units the life threatening issues take a priority and Nutrition takes a backseat. Nutrition is a therapy as important as others and influences the outcome of CCU patients. Early initiation of enteral nutrition (“Work the gut”) is the mainstay of nutrition in critical care. Owing to their disease process these patients are at an increased risk of being underfed and if not monitored properly they experience “Calorie Debt” with serious consequences on the outcome quantified using indicators like morbidity, mortality, stress ulcers, pressure ulcers and other markers.

## Objectives

To find the causes of interruption in enteral nutrition in adult patients admitted to the CCU of a tertiary care hospital in India.

## Methods

A prospective observational study was conducted over a period of 100 days in 3 mixed CCUs. On routine ICU rounds 200 patients on enteral nutrition were observed. Patients on parenteral nutrition were excluded. Patients shifted out of ICU were not followed up.

## Results

200 patients were observed twice a day, with 400 instances of observations, findings were instances of feed interruption 49 (12.25%) hemodynamic instability, 34.5%) acute GI Hemorrhage, 37 (9.25%) increased risk for aspiration, 9 (2.25%) Intestinal obstruction and 21 (5.25%) operational faults (kitchen service, pharmacy service and staff crisis) and on 250 (62.5%) instances patients received enteral nutrition at right time and same was maintained throughout their stay in the CCU. The identified causes of delay in initiation and maintenance of early enteral nutrition in critically ill were classified as avoidable and unavoidable. As a team the target was to initiate early enteral nutrition (within 24-48 hours of CCU admission) because of its pronounced physiological, infection control and financial benefits over parenteral nutrition and also because of the nutritional state of a patient significantly affects the outcome of the patient, avoidable causes must be strictly monitored, avoided, staff education and awareness must be spread amongst care givers. The “calorie debt” should be properly documented and the target at least 50-65% of calorie requirement must be made up within7 days of CCU admission or hospital stay.

## Conclusions

Enteral nutrition is vital but often overlooked in the face of conventional intensive care but care must be taken to minimize the avoidable causes.
